# Implementation and Effectiveness of Guideline-Recommended Clinical Activities for Children With Asthma: Population-Based Cohort

**DOI:** 10.1016/j.chest.2024.10.036

**Published:** 2024-11-07

**Authors:** Zainab Khalaf, Sejal Saglani, Chloe I. Bloom

**Affiliations:** National Heart and Lung Institute, Imperial College London, London, England

**Keywords:** asthma action plan, asthma management plan, asthma review, childhood asthma, exacerbations, guideline recommendations, inhaler technique

## Abstract

**Background:**

Guidelines advise minimizing asthma exacerbation risk, which is achieved partially through good clinical practice activities, including scheduled asthma reviews, inhaler technique checks, and asthma management plans. We assessed how frequently these activities are provided and how effective they are in clinical practice.

**Research Question:**

Do guideline-recommended activities such as asthma reviews, inhaler technique checks, and asthma management plans prevent asthma exacerbations?

**Study Design and Methods:**

This retrospective chart review used United Kingdom primary care medical records between 2004 and 2021, linked to hospital records. Children were eligible from asthma diagnosis until age 16 years. Annual implementation of asthma review, inhaler technique check, and asthma management plan was measured. Risk factors for these activities not being undertaken were determined by using multivariable logistic regression. Self-controlled case series was adopted to assess the effectiveness of each activity over 12 months; this was divided into two 6-month periods.

**Results:**

A total of 126,483 children were eligible; 30% to 45% received each annual activity, and 8% received all three together. Risk factors for not receiving activities included younger age, more socioeconomic deprivation, and higher or no BMI measurement. Management plans and asthma reviews, as standalone activities, were associated with an approximately 15% exacerbation reduction over 12 months and 8% over 6 months, respectively (asthma management plan, n = 4,624; 0-180 days [incidence rate ratio (95% CI)]: 0.87 [0.79-0.96]; 181-365 days: 0.83 [0.73-0.95]; asthma review, n = 6,948; 0-180 days: 0.92 [0.85-0.99]; 181-365 days: 0.93 [0.83-1.03]). Standalone inhaler technique checks were not associated with exacerbations. Provision of all activities together was associated with an approximately 30% exacerbation reduction over 12 months (n = 3,643; 0-180 days: incidence rate ratio, 0.76 [0.68-0.85]; 181-365 days: incidence rate ratio, 0.69 [95% CI, 0.60-0.81]).

**Interpretation:**

Our results indicate that most children in the United Kingdom do not receive the guideline-recommended activities to monitor their asthma. This study suggests that these activities, if implemented, are effective in clinical practice and maximally effective when combined in the same visit.

**Take-home Points**

**Study Question:** How often are routinely recommended good clinical practice activities implemented in children with asthma, and how effective are they?

**Results:** We found that annual asthma reviews, inhaler technique checks, and asthma management plans are often not implemented, especially in children who are younger, from more deprived areas, are obese, or who have no documentation of weight; these activities reduced asthma exacerbations, however.

**Interpretation:** Our results show that when implemented together in one consultation, these activities reduced asthma exacerbations by 30%, and should be implemented more frequently. The children most at-risk of exacerbations were least likely to receive them, however.

Asthma is a major cause of morbidity and mortality among children globally, and optimizing management strategies can significantly improve outcomes. In this respect, in addition to pharmacologic management, several clinical practices are recommended within national and international guidelines. These include regular dedicated scheduled asthma reviews, adherence monitoring, inhaler technique education, education on trigger avoidance, and provision of an asthma management/action plan.^1^, ^2^, ^3^

Unfortunately, questionnaires and medical record audits of primary care practices in the United States, Canada, Scotland, and England suggest widespread suboptimal implementation of asthma guideline recommendations.^4^, ^5^, ^6^, ^7^ The reasons for poor implementation are numerous, such as lack of resources and knowledge, as well as limited willingness based on perceived lack of benefit for patients.^5^^,^^6^ Furthermore, certain population groups are at greater risk of not receiving guideline recommendations. For example, for adults in the United States and the United Kingdom, there are significant differences in asthma care depending on where you live in relation to region or local health care infrastructure.^7^^,^^8^

Although we know that poor inhaler technique, reduced adherence, and lack of asthma education are associated with increased health care use and exacerbation risk, suboptimal interventions to improve these issues infer minimal change to long-term outcomes.^9^ A Cochrane review of 29 randomized controlled trials (RCTs), including adults and children, to improve inhaler technique found that most single or isolated interventions did not lead to sustained improvements in clinical outcomes.^10^ In addition, the effectiveness of asthma management plans seems to be related to the degree of associated asthma education as well as the intensity of clinical reviews.^11^

In the United Kingdom, > 95% of scheduled asthma care is provided by primary care.^12^ We therefore leveraged a nationwide database of primary care medical records to determine the implementation of three guideline-recommended good clinical practice activities (GCPAs): asthma reviews, asthma management plans, and inhaler technique checks. We determined which children were less likely to receive GCPAs and their effectiveness at reducing exacerbations.

## Study Design and Methods

### Data Source

The study population was drawn from a database of United Kingdom primary care medical records, the Clinical Practice Research Datalink (CPRD), the largest longitudinal health care database globally, covering approximately 20% of the United Kingdom.^13^ CPRD data have been found to be broadly nationally representative of the United Kingdom population in terms of age, sex, and ethnicity. Records were individually linked to English hospital admission and emergency department data through Hospital Episode Statistics.

### Ethical Approval

This study is based in part on data from the CPRD obtained under licence from the United Kingdom Medicines and Healthcare Products Regulatory Agency. The data are provided by patients and collected by the National Health Service as part of their care and support. The protocol for this research was approved by CPRD’s Research Data Governance Process (protocol number: 20_053).

Institutional review board approval was not required because the study did not involve human or animal participants or patients.

### Study Population and Design

**Figure 1:**
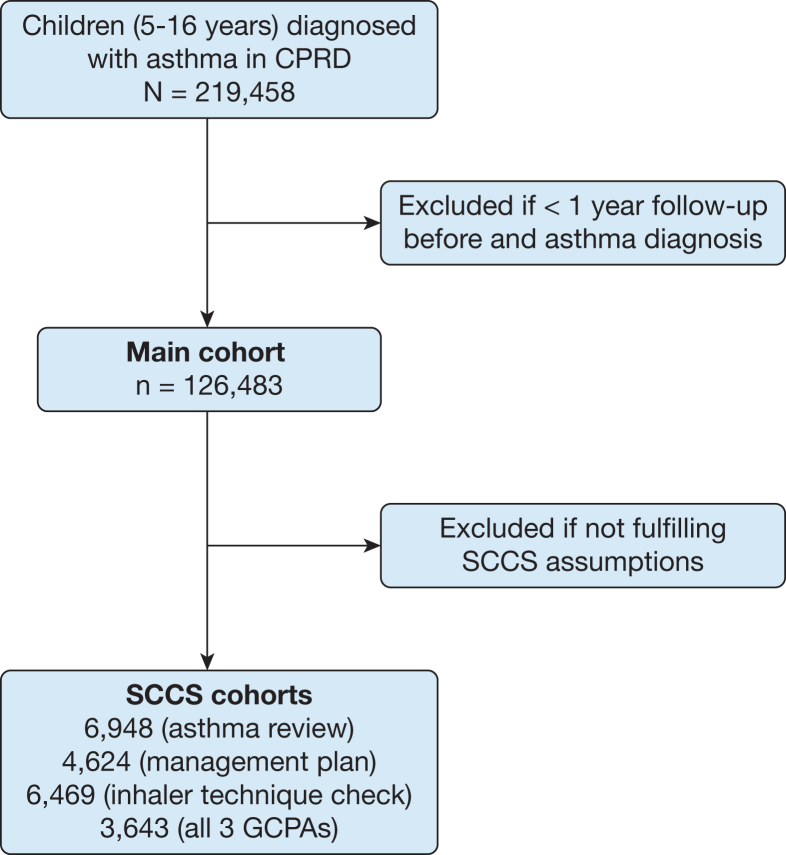
Flow diagram of the study. CPRD = Clinical Practice Research Datalink; GCPA = good clinical practice activity; SCCS = self-controlled case series.

The study included children (aged 5-16 years) identified between January 2004 and January 2021 who had at least three asthma codes (e-Table 1) within 2 years and a minimum of 1 year of data prior to entering the study and ≥ 12 months’ follow-up (Fig 1). Follow-up was censored at the earliest of the following: end of the study (January 1, 2022), leaving the general practitioner practice, date of death, or 18th birthday. The date of asthma diagnosis was defined as the first recorded asthma code.

**Figure 2:**
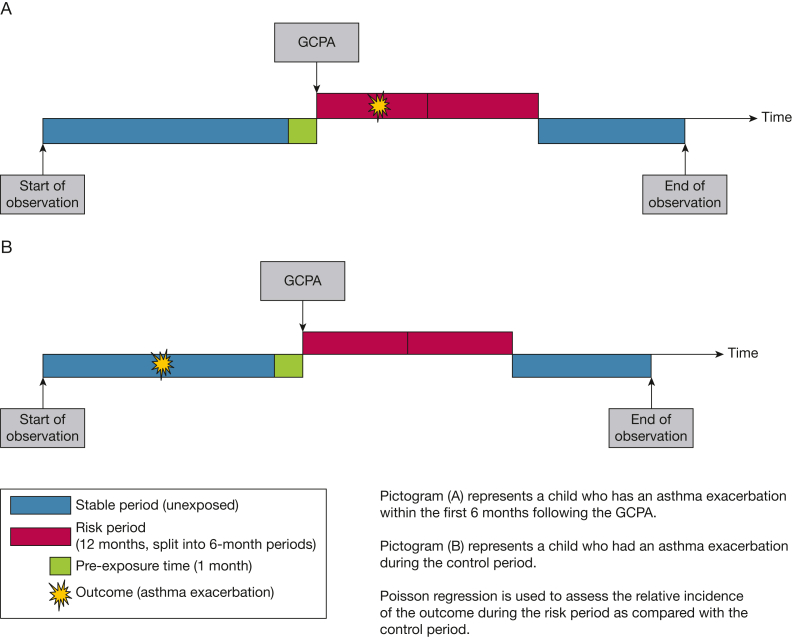
Self-controlled case series study design. GCPA = good clinical practice activity.

To evaluate the effectiveness of each GCPA, self-controlled case series (SCCS) methodology was used.^14^ The unique advantage of SCCS is that it completely mitigates any confounding that is constant throughout the observation period (eg, genetics and health care behavior). This is achieved as each child acts as their own control, and outcome events are compared prior to and following the exposure. Thus, only children receiving the GCPA and experiencing the outcome were eligible. There are several assumptions that must be met to ensure valid and unbiased estimates. First, recurrences of the outcome should be independent; to avoid violation of this assumption, therefore, only the first ever asthma exacerbation was included. Second, the outcome should not change the probability of exposure. If the outcome only likely affects the risk of the exposure within a certain time frame, this potential bias can be removed by using a “pre-exposure period,” which is excluded from the analysis. Because an asthma exacerbation could potentially lead to provision of a GCPA, a 31-day pre-exposure period was included (Fig 2). Third, the outcome should not censor the observation period (eg, asthma-related death); this rare outcome did not occur in the current cohort.

### Exposures

We addressed three guideline-recommended GCPAs: asthma review, inhaler technique check, and asthma management plan (e-Table 2).^1^^,^^2^^,^^15^ Definition of an asthma review was as follows: in the United Kingdom during the study period, to receive financial reward, a primary care asthma review had to be annual and had to include an assessment of asthma control, but no other stipulations were made.^16^ A GCPA was considered to be implemented on the day a code for that GCPA was used in the medical record. If no code was input, it was assumed the activity did not occur.

### Variables and Outcome

BMI was categorized by using *Z* scores derived from 1990 United Kingdom data according to World Health Organization standards.^17^ Socioeconomic status was assessed by using quintiles of the index of multiple deprivation, with quintile five being the highest relative level of deprivation. Smoking status was considered for both passive and active smoking. Three atopy-related variables (hay fever, food or drug allergy, and eczema) were considered present if recorded at least once. Asthma medications included short-acting beta-agonists, leukotriene receptor antagonists, and inhaled corticosteroids (ICSs), with dosages categorized as per 2019 British Thoracic Society/Scottish Intercollegiate Guidelines Network guidelines.^3^

An asthma exacerbation was defined as a short course of oral corticosteroids, emergency department visit, or hospital admission for asthma (International Classification of Diseases, 10th Revision codes J45 and J46).

### Statistical Analysis

The annual prevalence of each GCPA was calculated by dividing the number of children receiving at least one of that GCPA by the number of children with a complete year of data for each of the 12 months following the patient’s asthma diagnosis.

The association between children’s characteristics and receiving each GCPA, in the first year following diagnosis, was estimated by using three separate multivariable logistic regression models (one for each GCPA). Models were adjusted for age, sex, socioeconomic status, BMI, hay fever, food/drug allergy, eczema, smoking, and respiratory symptoms, exacerbations, and asthma medication in the year before. Several sensitivity analyses were conducted, including: (1) applying multinomial multivariable logistic regression and categorizing GCPA frequency in the first 3 years following an asthma diagnosis as never occurring, occurring just once, or occurring in at least 2 of 3 years; (2) imputing missing BMI values by using chained equations over 10 data sets with pooling results per Rubin’s rules; and (3) using a mixed effect model to address potential clustering according to general practitioner practice.

To evaluate the association between each GCPA and asthma exacerbations, we used four SCCS models, one for each of the three GCPAs and one for children who had received all three GCPAs during the same consultation. Children in the model receiving all three GCPAs could not be in the model for one GCPA alone. A fixed effect conditional Poisson model was used to derive incidence rate ratios (IRRs), comparing the control period (12 months prior to the GCPA) and the risk period (12 months following the GCPA). To examine for longitudinal effects, the risk period was segmented into two periods: 0 to 181 days, and 182 to 365 days following the GCPA. Models were adjusted for age, the time-varying confounder.

All analyses were conducted in Stata version 17 (Stata Corp).

## Results

### Prevalence of Receiving GCPAs

**Figure 3:**
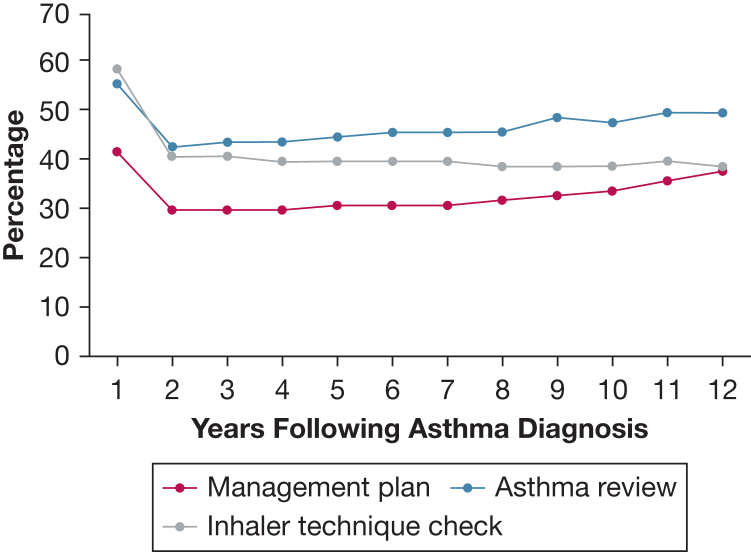
Annual prevalence of receiving each good clinical practice activity according to years since asthma diagnosis.

A total of 126,483 children were included in the study with mean ± SD follow-up of 4.3 ± 3.0 years. In the first-year following the asthma diagnosis, 56% received an annual asthma review, but this declined to approximately 45% in subsequent years (Fig 3). Asthma management plans were documented as being provided to 42% of children in the first year following diagnosis, and then dropping to around 30% thereafter. Inhaler technique checks were reported as conducted for 59% of children in the first year following diagnosis, but this too decreased to approximately 40%. Notably, 11,457 children (9%) received any of two GCPAs on the same day, and 10,035 (8%) received all three GCPAs on the same day in the first year following diagnosis.

### Association Between Characteristics and Continuous Receipt of GCPAs

Children who received a GCPA were slightly older, more likely to be male, were more likely to have a recorded BMI, were more likely to have a higher proportion with respiratory symptoms, and more likely to have received ICS prescriptions. Atopy and previous exacerbations, however, did not distinguish those who did and did not receive a GCPA (Table 1).

**Table 1:** Demographic and Clinical Characteristics of the Cohort

Characteristic	All Children	Asthma Review	Inhaler Technique Check	Management Plan	No Activity
Total	126,483 (100%)	72,001 (57%)	76,309 (60%)	54,484 (43%)	29,008 (23%)
Age, mean ± SD, y	9.7 ± 3.4	9.8 ± 3.4	9.9 ± 3.4	9.7 ± 3.4	8.4 ± 3.3
Male	68,229 (54%)	38,490 (53%)	40,587 (53%)	29,073 (53%)	12,847 (44%)
BMI					
Normal	56,006 (44%)	36,143 (50%)	40,141 (53%)	30,086 (55%)	7,668 (26%)
Overweight	12,291 (10%)	7,899 (11%)	8,702 (11%)	6,515 (12%)	1,707 (6%)
Obese	8,252 (7%)	5,123 (7%)	5,649 (7%)	4,284 (8%)	1,272 (4%)
Unknown	49,934 (39%)	22,836 (32%)	21,817 (29%)	13,599 (25%)	18,361 (63%)
IMD (socioeconomic status)					
1 (least deprived)	24,071 (19%)	14,346 (20%)	14,565 (19%)	9,903 (18%)	5,349 (18%)
2	22,755 (18%)	13,343 (18%)	13,873 (18%)	9,689 (18%)	5,078 (17%)
3	22,690 (18%)	12,927 (56%)	13,672 (18%)	9,533 (17%)	5,234 (18%)
4	26,587 (21%)	14,387 (20%)	15,814 (21%)	11,713 (21%)	6,302 (22%)
5	30,380 (24%)	16,998 (24%)	18,385 (24%)	13,646 (25%)	7,045 (24%)
Hay fever	21,813 (17%)	12,456 (17%)	13,211 (17%)	9,537 (18%)	4,850 (17%)
Eczema	42,459 (34%)	24,496 (34%)	25,298 (33%)	18,856 (35%)	9,853 (34%)
Food/drug allergy	9,975 (8%)	5,669 (8%)	5,784 (8%)	4,509 (8%)	2,424 (8%)
Events in the year before					
Reported dyspnea	9,499 (8%)	5,783 (8%)	6,272 (8%)	4,495 (8%)	1,705 (6%)
Reported wheeze	15,148 (12%)	9,314 (13%)	9,860 (13%)	7,610 (14%)	2,757 (10%)
Reported cough	43,571 (34%)	25,725 (36%)	27,671 (36%)	18,850 (35%)	8,744 (30%)
ICS inhaler prescriptions					
None	90,083 (71%)	48,971 (68%)	52,681 (69%)	36,706 (71%)	22,405 (77%)
1-3	32,553 (26%)	20,608 (29%)	21,262 (28%)	15,972 (29%)	5,661 (20%)
4-9	3,605 (3%)	2,284 (3%)	2,242 (3%)	1,701 (3%)	700 (2%)
≥ 10	242 (< 1%)	138 (< 1%)	124 (< 1%)	105 (< 1%)	242 (1%)
SABA inhaler prescriptions					
None	49,370 (39%)	25,201 (35%)	26,792 (35%)	18,353 (34%)	14,443 (49%)
1-3	69,218 (55%)	41,933 (58%)	44,637 (58%)	32,370 (59%)	13,008 (45%)
4-9	7,327 (5%)	4,537 (6%)	4,545 (6%)	3,497 (6%)	1,425 (5%)
≥ 10	568 (1%)	330 (1%)	335 (1%)	265 (1%)	132 (1%)
≥1 LTRA prescription	3,008 (2%)	1,849 (3%)	1,691 (2%)	1,464 (3%)	687 (2%)
Exacerbations					
None	118,272 (94%)	67,180 (94%)	71,399 (94%)	50,677 (93%)	27,098 (94%)
GP managed	6,625 (5%)	3,926 (5%)	4,053 (5%)	3,067 (6%)	1,484 (5%)
Hospital managed	1,586 (1%)	895 (1%)	857 (1%)	740 (1%)	426 (1%)

GP = general practitioner; ICS = inhaled corticosteroid; IMD = Index of Multiple Deprivation; LTRA = leukotriene receptor agonist; SABA = short-acting beta-agonist.

**Figure 4:**
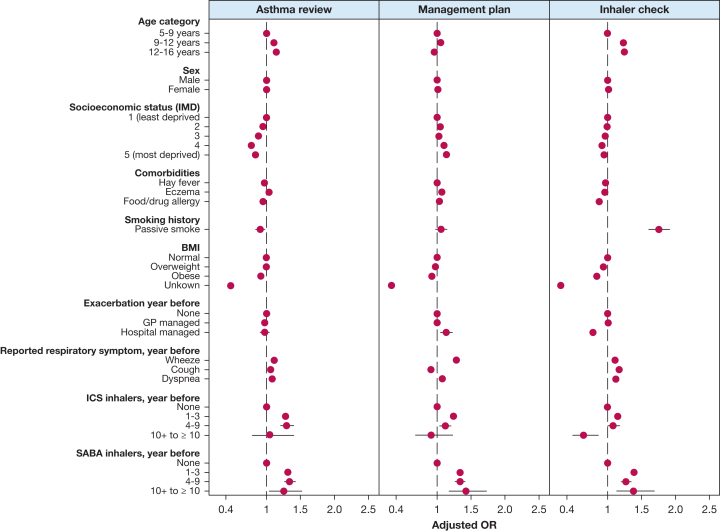
Association between children’s baseline characteristics and receiving each good clinical practice activity within 1 year of asthma diagnosis. GP = general practitioner; ICS = inhaled corticosteroid; IMD = Index of Multiple Deprivation; SABA = short-acting beta-agonist.

Factors significantly associated with not receiving a GCPA were younger age, lower socioeconomic status, not having BMI recorded, obesity, and not receiving inhaler prescriptions (e-Tables 3-5, Fig 4). For example, children aged > 12 years had a 25% increased odds of receiving an inhaler technique check compared with those aged 5 to 9 years (OR, 1.25; 95% CI, 1.22-1.29). Children residing in the most deprived areas had 16% reduced odds (95% CI, 0.81-0.87) of receiving an annual asthma review and 5% reduced odds (95% CI, 0.91-0.98) of an inhaler technique check but had 14% higher odds (95% CI, 1.10-1.18) of receiving an asthma management plan. Children without a recorded BMI had approximately 60% reduced odds of receiving any GCPA. Those with BMI categorized as obese had slightly reduced odds of receiving an annual asthma review, inhaler technique check, or asthma management plan (OR, 0.92 [95% CI, 0.87-0.96]; OR, 0.84 [95% CI, 0.80-0.88]; OR, 0.92 [95% CI, 0.88-0.97], respectively). Children prescribed ICSs or short-acting beta-agonist inhalers were more likely to receive an annual asthma review, inhaler technique check, and asthma management plan.

Sensitivity analyses found similar changes in the estimates. These included analyses to address factors influencing receiving GCPAs over a longer period (3 years) (e-Tables 6-8), adjustments for potential effects due to clinical differences between general practitioner practices, and inclusion of imputation for missing BMI values.

### Effectiveness of Each GCPA on Reducing Exacerbation Risk

The following numbers of children were eligible for each of the four SCCS models: 6,948 for asthma annual review, 6,469 for inhaler technique check, 4,624 for asthma management plan, and 3,643 for all three GCPAs during the same consultation (e-Table 9).

Standalone annual asthma reviews were associated with a 9% significant decrease in exacerbations only during the first 6 months (0-180 days: IRR, 0.92 [95% CI, 0.85-1.00; *P* = .034]; 181-365 days: IRR, 0.93 [95% CI, 0.83-1.03, *P* = .161]) (Table 2).

**Table 2:** Association Between Each GCPA as a Standalone Activity, and When Provided in a Single Consultation, With Asthma Exacerbations in the 12 Months Following Diagnosis

GCPA	Exposure Period (d)	Events (n)	Age-Adjusted IRR (95% CI)	*P* Value
Asthma review	1-180	2,522	0.92 (0.85-0.99)	< .05
	181-365	1,324	0.93 (0.83-1.03)	.161
Inhaler technique check	1-180	2,337	0.92 (0.84-1.00)	.065
	181-365	1,211	0.92 (0.82-1.03)	.174
Asthma management plan	1-180	1,653	0.87 (0.79-0.97)	< .01
	181-365	849	0.83 (0.73-0.97)	< .01
All three activities in same consultation	1-180	1,187	0.76 (0.68-0.85)	< .0001
	181-365	611	0.69 (0.60-0.81)	< .0001

GCPA = good clinical practice activity; IRR = incidence rate ratio.

Standalone inhaler technique checks were not associated with a significant decrease in exacerbations. There was, however, a small nonsignificant decrease (0-180 days: IRR, 0.92 [95% CI, 0.84-1.00, *P* = .065]; 181-365 days: IRR, 0.92 [95% CI, 0.82-1.03; *P* = .174]) (Table 2).

Standalone provision of an asthma management plan was associated with an approximate 15% significant decrease in exacerbations. This finding was persistent across the 12 months following the activity (0-180 days: IRR, 0.87 [95% CI, 0.79-0.97; *P* < .01]; 181-365 days: IRR, 0.83 [95% CI, 0.73-0.97; *P* < .01]) (Table 2).

Provision of all three GCPAs during the same consultation was associated with an approximate 30% decrease in exacerbations, persistent across the 12 months following the activities (0-180 days: IRR, 0.76 [95% CI, 0.68-0.85; *P* < .0001]; 181-365 days: IRR, 0.69 [95% CI, 0.60-0.81; *P* < .0001]) (Table 2).

## Discussion

In this United Kingdom study of children with asthma, nearly one-half did not receive a guideline-recommended GCPA for asthma, and only 8% received all three GCPAs in one consultation in their first year following diagnosis. Despite the relatively low implementation of GCPAs, we found that these activities were associated with a reduction in the risk of future asthma exacerbations. Their effectiveness was paramount when implemented altogether in one comprehensive consultation.

The most vulnerable at-risk children were the least likely to receive the activities, including younger children, those who were overweight or obese, and those of lower socioeconomic status; all of these variables are risk factors for worse outcomes.^18^ The group of children least likely to receive a GCPA were those without a BMI recorded in their medical records. This may be related to both reduced engagement with health care providers from the child’s caregiver or from suboptimal clinical practices by their general practitioner practice.

It is notable that the proportion of children who received an annual asthma review was closely aligned with the minimum threshold requirement of 45% that United Kingdom primary care practices must achieve to benefit from financial incentives, as part of the National Health Service’s performance management system (Quality Outcomes Framework).^19^ Asthma management plans and inhaler technique checks do not explicitly benefit from financial incentives, which may explain why fewer children (around 30%-40%) received these. The benefit of the United Kingdom financial reward system for patients with chronic conditions is unclear, and as threshold requirements do not stipulate which children should receive these clinical activities, the threshold potentially further aggravates health care disparities in asthma management.^19^^,^^20^

We also wished to understand how effective GCPAs are in real-life clinical practice; in particular, there is a distinct lack of evidence regarding the effectiveness of annual asthma reviews for children. A systematic review, including three RCTs (1999-2004, two from Australia and one from the United Kingdom) found there was no benefit from primary care asthma clinics in terms of hospitalizations, medication utilization, or overall quality of life; only one trial included children, however.^21^ A more recent, real-world US cohort study of 5,656 children found that well-child care visits were associated with a reduction in exacerbations.^22^ Well-child care visits could vary between practices but were designed to include an assessment of asthma control, education, and self-management support. The visits are therefore more comprehensive than the annual asthma reviews of the United Kingdom, which only specify a review of asthma control.^16^ Similar to our findings, the US study found that children at greatest risk of exacerbations were least likely to attend the well-child care visits.

United Kingdom asthma management guidelines state that because there is no evidence of harm from asthma reviews, they continue to recommend them.^3^ However, it could be argued that if blanket annual asthma reviews are ineffective, this costly clinical practice should be re-evaluated. The current study found that asthma reviews did reduce exacerbations, but the magnitude of effect was small, and potentially the effect was not sustained for > 6 months. However, when the review was combined with an inhaler technique check and an asthma management plan, there was a considerable reduction (approximately 30%) during the next 12 months. As in our study, the asthma review only had to involve assessment of asthma control (using a validated asthma control questionnaire); it is perhaps not surprising that this singular intervention was limited in its ability to negate adverse outcomes.

We also found that standalone inhaler technique checks had a minimal effect on exacerbations, which may be surprising as poor technique is strongly associated with suboptimal asthma control and exacerbations.^9^ However, our finding parallels a Cochrane review of 29 RCTs.^10^ The review assessed interventions to improve inhaler technique and addressed a wide variety of interventions. The review found that although some interventions resulted in a benefit to asthma control, they were not usually sustained and did not lead to significant clinical benefits. The evidence for children was less conclusive as the trials including children were fewer in both number and size.

We found that most benefit was gained from standalone asthma management plans, with approximately a 15% reduction in exacerbations for at least 12 months. A large meta-review of 27 systematic reviews and 13 updated RCTs found that supported self-management reduces hospitalization and improves asthma control and quality of life.^23^ For most trials, this included three elements: education, a management plan, and professional review. Trials were additionally maximally effective when encompassing proactive long-term asthma care. Evidence from these trials and our real-life clinical data together suggest that asthma management plans are effective but provide most benefit when combined with other asthma care practices.

Our real-world evidence, and the limited evidence regarding children that is available from RCTs, find that there is a greater improvement in asthma outcomes for children when interventions are delivered as a comprehensive package, including regular review, patient education, and supported self-management.^23^^,^^24^ Trials often do not replicate what happens in day-to-day clinical practice or real-life behavior as many patients with asthma are not eligible for trials (including children), many patients with asthma do not want to partake in trials, and trial conditions are optimized.^24^ Therefore, the agreement between our population-level real-life clinical practice data and the limited RCT data substantiates current guideline recommendations.

The current study has some key strengths. We believe this is the first nationwide assessment of these asthma clinical practices in children. By using routinely recorded data, the findings did not suffer from recall or selection bias. In addition, the self-controlled case series design removed confounding that did not change over the 2-year observation period (eg, health care behavior). The current study also had limitations. We do not know the details of what was included in each intervention. For example, some asthma reviews may have included an inhaler technique check. However, this was not part of the financial incentive, and we do not know how detailed or well conducted the activity was. It is possible some children received GCPAs in secondary care, but these numbers are likely to be low as most scheduled United Kingdom asthma care occurs in primary care. We had missing values for BMI, but our sensitivity analysis using multiple imputation found negligible differences. Furthermore, missing BMI data were unlikely to affect the SCCS findings because BMI was unlikely to change much over the observation period. We did not evaluate recurrent exacerbations. We were unable to evaluate outcomes other than exacerbations due to the data available. For example, we did not have questionnaire data on asthma control, school absenteeism, or quality of life. Finally, because this study was restricted to the United Kingdom, we may not be able to generalize the findings to other health care systems.

## Interpretation

This study found that just more than one-half of children in the United Kingdom do not receive asthma guideline recommendations: asthma reviews, inhaler technique checks, or asthma management plans. Those at the highest risk of not receiving them were the children at greatest risk of poor asthma outcomes, including children from lower socioeconomic backgrounds, with excess BMI or no BMI recording, younger children, and those with excess inhaler prescriptions. We found these activities to be effective in reducing asthma exacerbations in real-life clinical practice. Receiving all 3 activities in one consultation had the greatest benefit, reducing the risk of an exacerbation in the following year by 30%. This study highlights the need for a more integrated and inclusive approach. We need to find new ways to engage with at-risk children and their caregivers to encourage a better relationship with health care professionals and implementation of proactive, preventative measures.

## Funding/Support

This study was supported by the National Heart and Lung Institute Foundation Centre for Airways Disease in Children & Young Adults and the 10.13039/501100000272National Institute for Health Research Imperial Biomedical Research Centre.

## Financial/Nonfinancial Disclosures

The authors have reported to *CHEST* the following: C. I. B. receives support from the National Institute for Health and Care Research and Asthma and Lung UK, outside the submitted work. None declared (S. S., Z. K.).

## Supplementary Data

e-Online Data
