# Supplementary material for: Implementation and Effectiveness of Guideline-Recommended Clinical Activities for Children With Asthma: Population-Based Cohort
Source: Chest. 2024 Nov 7;167(3):665–74. doi: 10.1016/j.chest.2024.10.036 (PMC11882771; doi:10.1016/j.chest.2024.10.036)
Supplement: e-Online Data [file mmc1.docx]

**Supplementary tables and figures**

Supplementary table E1: Asthma codes


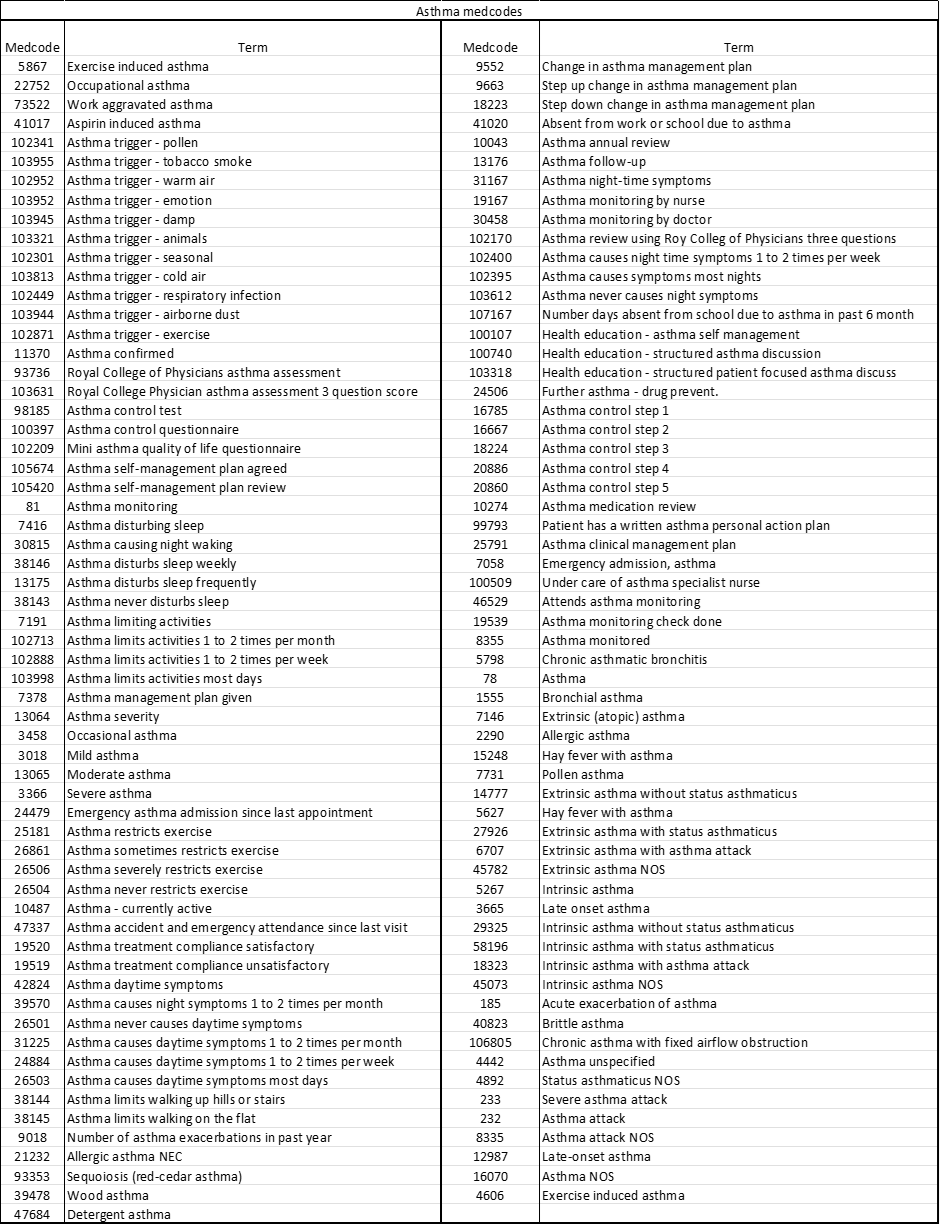


Supplementary table E2: Good Clinical practice Activities codes

| **Asthma reviews** | |
| --- | --- |
| Medcode | Term |
| 1488421017 | Asthma annual review |
| 1488436019 | Asthma medication review |
| 1753401000006117 | Asthma review using Royal College of Physicians three questions |
| 7518271000006114 | Did not attend asthma review |
| **Asthma management plan** | |
| 2115631000000117 | Asthma self-management plan review |
| 264550017 | Asthma management |
| 1484905010 | Change in asthma management plan |
| 1484911013 | Step down change in asthma management plan |
| 2474332015 | Asthma clinical management plan |
| 235661000000118 | Does not have asthma management plan |
| 1176501000000114 | Health education - asthma self management |
| 1880001000006114 | No change in asthma management plan |
| 2117771000000117 | Asthma self-management plan agreed |
| 2297651000000118 | Asthma management plan declined |
| 7510501000006115 | Education about asthma self management |
| 7688501000006112 | Recent asthma management |
| 12734731000006116 | Registration for access to online asthma self-management application |
| **Inhaler check** | |
| 264515013 | Demonstration of inhaler technique |
| 264516014 | Inhaler technique observed |
| 264534013 | Inhaler technique - good |
| 264535014 | Inhaler technique - poor |
| 1484902013 | Inhaler technique - moderate |
| 3518260015 | Inhaler technique demonstrated by patient |
| 855741000006114 | Inhaler technique |
| 1658981000000111 | Provision of written information on inhaler technique |
| 2264951000000115 | Review of inhaler technique using inhaler checking device |
| 8295311000006117 | Check of inhaler technique not appropriate |

|  | **Adjusted odds ratio** | **p-value** | **95% confidence interval** | |
| --- | --- | --- | --- | --- |
| **Age category (years)** | | | | |
| 5-9 | *Reference* | | | |
| 9-12 | 1.10 | <0.001 | 1.07 | 1.13 |
| 12-16 | 1.14 | <0.001 | 1.10 | 1.18 |
| **Gender** |  |  |  |  |
| Female | 1.00 | 0.569 | 0.98 |  |
| **IMD (socioeconomic status)** | | | | |
| 1 (least deprived) | *Reference* | | | |
| 2 | 0.95 | 0.005 | 0.91 | 0.98 |
| 3 | 0.89 | <0.001 | 0.85 | 0.92 |
| 4 | 0.78 | <0.001 | 0.75 | 0.80 |
| 5 | 0.84 | <0.001 | 0.81 | 0.87 |
| **Hay fever** | 0.96 | 0.050 | 0.93 | 0.99 |
| **Eczema** | 1.04 | 0.003 | 1.01 | 1.06 |
| **Food/drug allergy** | 0.95 | 0.016 | 0.90 | 0.98 |
| **Passive smoking** | 0.90 | 0.014 | 0.83 | 0.98 |
| **BMI** |  |  |  |  |
| Normal | *Reference* | | | |
| Overweight | 0.99 | 0.820 | 0.95 | 1.03 |
| Obese | 0.92 | <0.001 | 0.87 | 0.96 |
| Unknown | 0.47 | <0.001 | 0.46 | 0.48 |
| **Year before study start** | | | | |
| **Exacerbation** | | | | |
| None | *Reference* | | | |
| GP managed exacerbation | 0.97 | 0.249 | 0.90 | 0.99 |
| Hospital managed exacerbation | 0.97 | 0.407 | 0.90 | 1.04 |
| **Reported respiratory symptoms** | | | | |
| Wheeze | 1.11 | <0.001 | 1.07 | 1.15 |
| Cough | 1.06 | <0.001 | 1.03 | 1.09 |
| Dyspnoea | 1.08 | 0.001 | 1.03 | 1.13 |
| **ICS inhalers** | | | | |
| None | *Reference* | | | |
| 1-3 inhalers | 1.28 | <0.001 | 1.24 | 1.31 |
| 4-9 inhalers | 1.29 | <0.001 | 1.19 | 1.39 |
| 10+ | 1.04 | 0.02 | 1.03 | 1.52 |
| **SABA inhalers** | | | | |
| None | *Reference* | | | |
| 1-3 inhalers | 1.31 | <0.001 | 1,27 | 1..34 |
| 4-9 inhalers | 1.33 | <0.001 | 1.26 | 1.42 |
| 10+ | 1.25 | 0.020 | 1.03 | 1.52 |

Supplementary Table E3: Multivariable association between each baseline characteristic and receiving an annual asthma review within one year after asthma diagnosis (the model is adjusted for all variables shown)

IMD= index of multiple deprivation, BMI=body mass index, ICS=inhaled corticosteroids, SABA=short acting beta antagonist

Supplementary table E4: Multivariable association between each baseline characteristic and receiving an inhaler technique check within one year after asthma diagnosis (the model is adjusted for all variables shown)

|  | **Adjusted Odds ratio** | **p-value** | **95% confidence interval** | |
| --- | --- | --- | --- | --- |
| **Age category (years)** | | | | |
| 5-8.9 | *Reference* | | | |
| 9-11.9 | 1.23 | <0.001 | 1.20 | 1.27 |
| 12-16 | 1.25 | <0.001 | 1.22 | 1.29 |
| **Gender** |  |  |  |  |
| Female | 1.01 | 0.266 | 1.21 | 1.29 |
| **IMD** | | | | |
| 1 (least deprived) | *Reference* | | | |
| 2 | 0.99 | 0.804 | 0.96 | 1.03 |
| 3 | 0.97 | 0.092 | 0.93 | 1.00 |
| 4 | 0.92 | <0.001 | 0.88 | 0.95 |
| 5 | 0.95 | 0.006 | 0.91 | 0.98 |
| **Hay fever** | 0.96 | 0.040 | 0.93 | 0.99 |
| **Eczema** | 0.95 | 0.001 | 0.93 | 0.98 |
| **Food/drug allergy** | 0.88 | <0.001 | 0.84 | 0.91 |
| **Passive smoking** | 1.70 | <0.001 | 1.60 | 1.92 |
| **BMI** |  |  |  |  |
| Normal | *Reference* | | | |
| overweight | 0.94 | 0.006 | 0.90 | 0.98 |
| obese | 0.84 | <0.001 | 0.80 | 0.88 |
| unknown | 0.31 | <0.001 | 0.30 | 0.32 |
| **Year before study start** | | | | |
| **Exacerbation** |  |  |  |  |
| **None** | *Reference* | | | |
| GP managed exacerbation | 1.01 | 0.626 | 0.96 | 1.05 |
| Hospital managed exacerbation | 0.78 | <0.001 | 0.73 | 0.84 |
| **Reported respiratory symptoms** | | | | |
| Wheeze | 1.11 | <0.001 | 1.07 | 1.16 |
| Cough | 1.17 | <0.001 | 1.13 | 1.19 |
| Dyspnoea | 1.12 | <0.001 | 1.07 | 1.17 |
| **ICS inhalers** |  |  |  |  |
| None | *Reference* | | | |
| 1-3 | 1.15 | <0.001 | 1.11 | 1.18 |
| 4-9 | 1.01 | 0.050 | 1.00 | 1.17 |
| 10+ | 0.64 | 0.004 | 0.48 | 0.87 |
| **SABA inhalers** |  |  |  |  |
| None | *Reference* | | | |
| 1-3 | 1.39 | <0.001 | 1.35 | 1.42 |
| 4-9 | 1.27 | <0.001 | 1.20 | 1.35 |
| 10+ | 1.38 | 0.001 | 1.13 | 1.68 |

IMD= index of multiple deprivation, BMI=body mass index, ICS=inhaled corticosteroids, SABA=short acting beta agonist

Supplementary table E5: Multivariable association between each baseline characteristic and receiving a management plan within one year after asthma diagnosis (the model is adjusted for all variables shown)

|  | **Adjusted odds ratio** | **p-value** | **95% confidence interval** | |
| --- | --- | --- | --- | --- |
| **Age category (years)** | | | | |
| 5-8.9 | *Reference* | | | |
| 9-11.9 | 1.05 | 0.001 | 1.02 | 1.08 |
| 12-16 | 0.96 | 0.011 | 0.93 | 0.99 |
| **Gender** | | | | |
| Female | 1.01 | 0.001 | 1.02 | 1.08 |
| **IMD** | | | | |
| 1 (least deprived) | *Reference* | | | |
| 2 | 1.05 | 0.012 | 1.01 | 1.09 |
| 3 | 1.03 | 0.138 | .99 | 1.06 |
| 4 | 1.10 | <0.001 | 1.06 | 1.14 |
| 5 | 1.14 | <0.001 | 1.10 | 1.18 |
| **Hay fever** | 0.99 | 0.760 | 0.96 | 1.02 |
| **Eczema** | 1.06 | 0.000 | 1.04 | 1.09 |
| **Food/drug allergy** | 1.04 | 0.111 | 0.99 | 1.08 |
| **Passive smoking** | 1.06 | 0.161 | 0.97 | 1.14 |
| **BMI** | | | | |
| Normal | *Reference* | | | |
| Overweight | 0.97 | 0.228 | 0.93 | 1.01 |
| Obese | 0.92 | 0.001 | 0.88 | 0.97 |
| Unknown | 0.32 | 0.000 | 0.32 | 0.33 |
| **Year before study start** | | | | |
| **Exacerbation** | | | | |
| None | *Reference* | | | |
| GP managed | 0.99 | 0.233 | 0.93 | 1.01 |
| Hospital managed | 1.13 | 0.001 | 1.02 | 1.22 |
| **Reported respiratory symptoms** | | | | |
| Wheeze | 1.28 | <0.001 | 1.24 | 1.33 |
| Cough | 0.91 | <0.001 | 0.88 | 0.93 |
| Dyspnoea | 1.07 | <0.001 | 1.03 | 1.12 |
| **ICS inhalers** | | | | |
| None | Reference | | | |
| 1-3 | 1.24 | <0.001 | 1.21 | 1.28 |
| 4-9 | 1.12 | 0.003 | 1.03 | 1.21 |
| 10+ | 0.91 | 0.55 | 0.68 | 1.22 |
| **SABA inhalers** | | | | |
| None | *Reference* | | | |
| 1-3 | 1.34 | <0.001 | 1.30 | 1.37 |
| 4-9 | 1.33 | <0.001 | 1.26 | 1.42 |
| 10+ | 1.42 | <0.001 | 1.17 | 1.73 |

IMD= index of multiple deprivation, BMI=body mass index, ICS=inhaled corticosteroids, , SABA=short acting beta agonist

Supplementary table E6: Multinomial multivariable logistic regression model assessing each characteristic, comparing the frequency of receiving an annual asthma review (once, or 2-3 times) to children that did not receive an annual asthma review, during three year follow-up after asthma diagnosis

|  | **1 annual asthma review in 3 years** | | | **2-3 annual asthma reviews in 3 years** | | |
| --- | --- | --- | --- | --- | --- | --- |
|  | **Adjusted odd Ratio** | **p-value** | **95% CI** | **Adjusted odd Ratio** | **p-value** | **95% CI** |
| **Gender** |  | | |  | | |
| Female | 0.98 | 0.43 | (0.93-1.03) | 0.97 | 0.17 | (0.92-1.01) |
| **Age category (years)** |  | | |  | | |
| 5-8.9 | *Reference* | | | | | |
| 9-11.9 | 1.32 | <0.001 | (1.25-1.4) | 1.36 | <0.001 | (1.29-1.44) |
| 12-16 | 2.32 | <0.001 | (2.13-2.53) | 2.63 | <0.001 | (2.42-2.86) |
| **IMD (socioeconomic status)** | | | | | | |
| 1 (least deprived) | *Reference* | | | | | |
| 2 | 0.92 | 0.05 | (0.85-1) | 0.86 | <0.001 | (0.8-0.93) |
| 3 | 0.95 | 0.18 | (0.87-1.03) | 0.83 | <0.001 | (0.77-0.89) |
| 4 | 0.89 | 0.01 | (0.83-0.97) | 0.7 | <0.001 | (0.65-0.76) |
| 5 | 0.94 | 0.09 | (0.87-1.01) | 0.74 | <0.001 | (0.69-0.8) |
| **Hay fever** | 1.05 | 0.14 | (0.98-1.12) | 1.09 | 0.01 | (1.02-1.16) |
| **Eczema** | 0.96 | 0.14 | (0.91-1.01) | 1.01 | 0.76 | (0.96-1.06) |
| **Food/drug allergy** | 1.07 | 0.14 | (0.98-1.18) | 1.01 | 0.91 | (0.92-1.1) |
| **Passive smoking** | 0.91 | 0.28 | (0.77-1.08) | 0.88 | 0.139 | (0.75-1.04) |
| **BMI** |  | | |  | | |
| Normal | *Reference* | | | | | |
| Overweight | 1.08 | 0.15 | (0.97-1.21) | 0.99 | 0.82 | (0.89-1.1) |
| Obese | 1.22 | 0.003 | (1.06-1.39) | 0.98 | 0.77 | (0.86-1.11) |
| Unknown | 0.62 | <0.001 | (0.59-0.66) | 0.37 | <0.001 | (0.35-0.38) |
| Year before study start | | | | | | |
| **Exacerbation** | | | | | | |
| None | *Reference* | | | | | |
| GP managed | 1.06 | 0.22 | (0.97-1.16) | 1.05 | 0.213 | (1.00-1.17) |
| Hospital managed | 1.06 | 0.46 | (0.90-1.24) | 1.06 | 0.409 | (0.91-1.24) |
| **Reported respiratory symptoms** | | | | | | |
| Wheeze | 1.04 | 0.290 | (0.96-1.13) | 1.08 | 0.021 | (1.00-1.17) |
| Cough | 1.06 | 0.025 | (1.00-1.11) | 1.21 | <0.001 | (1.15-1.27) |
| Dyspnoea | 0.99 | 0.868 | (0.889-1.10) | 1.12 | 0.02 | (1.02-1.266) |
| **ICS inhalers** | | | | | | |
| None | *Reference* | | | | | |
| 1-3 | 1.28 | <0.001 | (1.20-1.36) | 1.35 | <0.001 | (1.27-1.43) |
| 4-10 | 1.35 | <0.001 | 1.15-1.59 | 1.47 | <0.001 | (1.25-1.73) |
| 10+ | 1.07 | 0.768 | (0.59-2.03) | 1.50 | 0.194 | (0.81-2.80) |
| **SABA inhalers** | | | | | | |
| None | *Reference* | | | | | |
| 1-3 | 1.52 | <0.001 | (1.44-1.98) | 1.52 | <0.001 | (1.44-1.60) |
| 4-9 | 1.60 | <0.001 | (1.42-1.81)) | 1.60 | <0.001 | (1.42-1.80) |
| 10+ | 1.58 | 0.032 | (1.04-2.41) | 1.58 | 0.03 | (1.04-2.40) |

IMD= index of multiple deprivation, BMI=body mass index, ICS=inhaled corticosteroids, SABA=short acting beta agonist

Supplementary table E7: Multinomial multivariable logistic regression model assessing each characteristic, comparing the frequency of receiving an annual inhaler technique check (once, or 2-3 times) to children that did not receive an annual asthma review, during three year follow-up after asthma diagnosis

|  | **1 Inhaler check in 3 years** | | | | | | **2-3 Inhaler check in 3 years** | | |
| --- | --- | --- | --- | --- | --- | --- | --- | --- | --- |
|  | **Adjusted odd Ratio** | **p-value** | | **95% confidence interval** | | | **Adjusted odd Ratio** | **p-value** | **95% confidence interval** |
| **Gender** |  | | | | | |  | | |
| Female | 0.96 | <0.001 | | (0.91-1.01) | | | 0.94 | 0.043 | (0.90-0.99) |
| **Age category (years)** |  | | | | | |  | | |
| 5-8.9 | *Reference* | | | | | | | | |
| 9-11.9 | 1.4 | <0.001 | | (1.33-1.51) | | | 1.40 | <0.001 | (1.32-1.49) |
| 12-16 | 2.3 | <0.001 | | (2.1-2.52) | | | 2.39 | <0.001 | (2.18-2.62) |
| **IMD (socioeconomic status)** | | | | | | | | | |
| 1 ( least deprived) | *Reference* | | | | | | | | |
| 2 | 0.95 | 0.21 | | (0.87-1.03) | | | 0.92 | 0.04 | (0.84-1.00) |
| 3 | 0.92 | 0.05 | | (0.85-1) | | | 0.83 | <0.001 | (0.76-0.90) |
| 4 | 0.92 | 0.06 | | (0.85-1) | | | 0.78 | <0.001 | (0.72-0.85) |
| 5 | 0.9 | 0.01 | | (0.83-0.97) | | | 0.76 | <0.001 | (0.70-0.82) |
| **Hay fever** | 1.03 | 0.4 | | (0.96-1.11) | | | 1.06 | 0.13 | (0.98-1.13) |
| **Eczema** | 0.97 | 0.23 | | (0.92-1.02) | | | 0.96 | 0.12 | (0.91-1.01) |
| **Food/Drug allergy** | 0.91 | 0.05 | | (0.83-1) | | | 0.86 | <0.001 | (0.78-0.95) |
| **Passive smoking** | 1.35 | <0.001 | | (1.11-1.65) | | | 1.53 | <0.001 | (1.26-1.87) |
| **BMI** | | | | | | | | | |
| Normal | *Reference* | | | | | | | | |
| Overweight | 1.01 | 0.83 | | (0.89-1.15) | | | 0.89 | 0.07 | (0.79-1.01) |
| Obese | 0.96 | 0.57 | | (0.83-1.11) | | | 0.73 | <0.001 | (0.63-0.84) |
| Unknown | 0.38 | <0.001 | | (0.36-0.4) | | | 0.19 | <0.001 | (0.18-0.21) |
| Year before study start | | | | | | | | | |
| **Exacerbation** | | | | | | | | | |
| None | *Reference* | | | | | | | | |
| GP managed | 1.02 | | 0.64 | | (0.92-1.14) | 1.00 | | 0.98 | (0.90-1.10) |
| Hospital managed | 0.87 | | 0.10 | | (0.75-1.02) | 0.83 | | 0.02 | (0.71-0.997) |
| **Reported respiratory symptoms** | | | | | | | | | |
| Wheeze | 1.14 | | <0.001 | | (1.04-1.26) | 1.2 | | <0.001 | (1.10-1.31) |
| Cough | 1.07 | | 0.01 | | (1.01-1.13) | 1.21 | | <0.001 | (1.14-1.27) |
| Dyspnoea | 1.07 | | 0.22 | | (0.95-1.20) | 1.24 | | <0.001 | (1.10-1.39) |
| **ICS inhalers** | | | | | | | | | |
| None | *Reference* | | | | | | | | |
| 1-3 | 1.11 | | <0.01 | | (1.04-1.19) | 1.22 | | <0.001 | (1.14-1.30) |
| 4-9 | 1.13 | | 0.18 | | (0.94-1.35) | 1.35 | | <0.01 | (1.13-1.60) |
| 10+ | 0.79 | | 0.46 | | (0.43-1.45) | 0.79 | | 0.42 | (0.44-1.41) |
| **SABA inhalers** | | | | | | | | | |
| None | *Reference* | | | | | | | | |
| 0-3 | 1.30 | <0.001 | | (1.22-1.37) | | | 1.52 | <0.001 | (1.42-1.63) |
| 4-10 | 1.15 | 0.03 | | (1.01-1.30) | | | 1.40 | <0.001 | (1.23-1.58) |
| 10+ inhalers | 1.00 | 0.02 | | (0,65-1.53) | | | 1.34 | <0.001 | (0.89-2.02) |

Supplementary table E8: Multinomial multivariable logistic regression model assessing each characteristic, comparing the frequency of receiving an annual asthma management plan (once, or 2-3 times) to children that did not receive an annual asthma review, during three year follow-up after asthma diagnosis

|  | **1 management plan in 3 years** | | | **2-3 management plans in 3 years** | | |
| --- | --- | --- | --- | --- | --- | --- |
|  | **Adjusted odd Ratio** | **p-value** | **95% confidence interval** | **Odd Ratio** | **p-value** | **95% confidence interval** |
| **Gender** | | | | | | |
| Female | 0.96 | 0.11 | (0.92-1.01) | 0.95 | 0.031 | (0.90-0.99) |
| **Age category (years)** | | | | | | |
| 5-8.9 | *Reference* | | | | | |
| 9-11.9 | 1.37 | <0.001 | (1.30-1.45) | 1.33 | <0.001 | (1.26-1.41) |
| 12-16 | 2.63 | <0.001 | (2.43-2.86) | 2.53 | <0.001 | (2.33-2.75) |
| **IMD (socioeconomic status)** | | | | | | |
| 1 (least deprived) | *Reference* | | | | | |
| 2 | 1.04 | 0.33 | (0.96-1.12) | 1.03 | 0.38 | (0.96-1.12) |
| 3 | 0.96 | 0.24 | (0.89-1.03) | 0.95 | 0.22 | (0.88-1.03) |
| 4 | 1.01 | 0.86 | (0.94-1.08) | 1.01 | 0.85 | (0.94-1.08) |
| 5 | 1.02 | 0.65 | (0.95-1.09) | 1.06 | 0.13 | (0.98-1.13) |
| **Hay fever** | 1.04 | 0.17 | (0.98-1.11) | 1.04 | 0.18 | (0.98-1.11) |
| **Eczema** | 0.97 | 0.19 | (0.92-1.02) | 1.00 | 0.98 | (0.95-1.05) |
| **Food/drug allergy** | 0.98 | 0.66 | (0.90-1.07) | 0.98 | 0.65 | (0.90-1.07) |
| **Passive smoking** | 1.12 | 0.16 | (0.95-1.32) | 0.97 | 0.73 | (0.82-1.15) |
| **BMI** | | | | | | |
| Normal | *Reference* | | | | | |
| Overweight | 1.06 | 0.28 | (0.96-1.17) | 0.99 | 0.85 | (0.90-1.09) |
| Obese | 1.02 | 0.79 | (0.90-1.14) | 0.87 | 0.02 | (0.77-0.97) |
| Unknown | 0.47 | <0.001 | (0.45-0.49) | 0.25 | <0.001 | (0.24-0.27) |
| **Year before study start** | | | | | | |
| **Exacerbation** |  |  |  |  |  |  |
| None | *Reference* | | | | | |
| GP managed | 1.08 | 0.06 | (0.99-1.17) | 1.02 | 0.55 | (0.94-1.11) |
| Hospital managed | 0.91 | 0.22 | (0.79-1.05) | 0.90 | 0.19 | (0.78-1.04) |
| **Reported respiratory symptoms** | | | | | | |
| Wheeze | 1.12 | 0.24 | (0.96-1.14) | 1.25 | <0.001 | (1.16-1.35) |
| Cough | 0.93 | <0.01 | (0.88-0.97) | 0.97 | 0.40 | (0.93-1.02) |
| Dyspnoea | 1.01 | 0.71 | (0.92-1.12) | 1.11 | 0.04 | (1.00-1.22) |
| **ICS inhalers** | | | | | | |
| None | *Reference* | | | | | |
| 1-3 | 1.13 | <0.001 | (1.07-1.20) | 1.20 | <0.001 | (1.13-1.27) |
| 4-10 | 1.20 | 0.02 | (1.03-1.39) | 1.30 | <0.001 | (1.12-1.51) |
| 10+ | 1.14 | 0.61 | (0.67-1.96) | 0.85 | 0.57 | (0.49-1.48) |
| **SABA inhalers** | | | | | | |
| None | *Reference* | | | | | |
| 1-3 | 1.17 | <0.001 | (1.11-1.23) | 1.41 | <0.001 | (1.33-1.48) |
| 4-9 | 1.08 | 0.09 | (0.98-1.22) | 1.36 | <0.001 | (1.22-1.52) |
| 10+ | 1.08 | 0.67 | (0.74-1.59) | 1.66 | <0.01 | (1.14-2.41) |

IMD= index of multiple deprivation, BMI=body mass index, ICS=inhaled corticosteroids, SABA=short acting beta agonist

Supplementary table E9: Descriptive analysis for the SCCS cohort for each activity

|  | **Asthma review**  **N (%)** | **Inhaler check**  **N (%)** | **Management plan**  **N (%)** |
| --- | --- | --- | --- |
| **Total** | 6,948 | 6,469 | 4,624 |
| **Age of diagnosis, years, mean (SD)** | 6.3 (3.6) | 6.3 (3.5) | 6.2 (3.5) |
| **Gender** |  |  |  |
| Males | 3,975 (57%) | 3,692 (57%) | 2,622 (57%) |
| **BMI** |  |  |  |
| Normal | 2,020 (29%) | 1,870 (29%) | 1,485 (32%) |
| Overweight | 372 (5%) | 347 (5%) | 262 (6%) |
| Obese | 218 (3%) | 236 (4%) | 166 (4%) |
| Unknown | 4,338 (62%) | 4,016 (62%) | 2,711 (59%) |
| **IMD (socioeconomic status)** |  |  |  |
| 1 | 1,247 (18%) | 1,119 (17%) | 724 (16%) |
| 2 | 1,250 (18%) | 1,114 (17%) | 776 (17%) |
| 3 | 1,241 (18%) | 1,114 (17%) | 813 (18%) |
| 4 | 1,458 (21%) | 1,441 (22%) | 1,027 (22%) |
| 5 (Most deprived) | 1,737 (25%) | 1,663 (26%) | 1,271 (27%) |
| Aeroallergen sensitization | 1,565 (23%) | 2,404 (37%) | 1,693 (37%) |
| Eczema | 2,688 (39%) | 2,930 (45%) | 2,172 (47%) |
| Food/drug allergy | 773 (11%) | 1,008 (16%) | 772 (17%) |
| Passive smoking | 294 (4%) | 514 (8%) | 325 (7%) |
| **In the year before GCPA** | | | |
| **Reported respiratory symptoms** | | | |
| Dysphonia | 441 (6%) | 1,121 (17%) | 798 (17%) |
| Wheeze/chest tightness | 978 (14%) | 2,275 (35%) | 1,708 (37%) |
| Cough | 1,695 (24%) | 4,923 (76%) | 3,514 (76%) |
| **SABA inhalers** |  |  |  |
| None | 1,318 (19%) | 1,213 (19%) | 821 (18%) |
| 1-3 inhalers | 4,250 (61%) | 3,947 (61%) | 2,848 (62%) |
| 4-9 inhalers | 1,267 (18%) | 1,191 (18%) | 871 (19%) |
| 10+ | 113 (2%) | 118 (2%) | 84 (2%) |
| **ICS inhalers** |  |  |  |
| None | 2,425 (35%) | 2,289 (35%) | 1,502 (32%) |
| 1-3 inhalers | 3,363 (48%) | 3,103 (48%) | 2,284 (49%) |
| 4-9 inhalers | 1,068 (15%) | 987 (15%) | 759 (16%) |
| 10+ inhalers | 92 (1%) | 90 (1%) | 79 (2%) |

IMD= index of multiple deprivation, BMI=body mass index, ICS=inhaled corticosteroids, SABA=short acting beta agonist
